# Soil organic carbon and total nitrogen pools in permafrost zones of the Qinghai-Tibetan Plateau

**DOI:** 10.1038/s41598-018-22024-2

**Published:** 2018-02-26

**Authors:** Lin Zhao, Xiaodong Wu, Zhiwei Wang, Yu Sheng, Hongbing Fang, Yonghua Zhao, Guojie Hu, Wangping Li, Qiangqiang Pang, Jianzong Shi, Bentian Mo, Qian Wang, Xirui Ruan, Xiaodong Li, Yongjian Ding

**Affiliations:** 1 Cryosphere Research Station on the Qinghai-Tibetan Plateau, State Key Laboratory of Cryospheric Science, Northwest Institute of Eco-Environment and Resources, Chinese Academy of Sciences, Lanzhou, 730000 China; 2 Guizhou Institute of Prataculture, Guizhou Academy of Agricultural Sciences, Guiyang, 550006 China; 3 School of civil engineering, Lanzhou University of Technology, Lanzhou, 730050 China; 4 Key Laboratory of Ecohydrology of River Basin Sciences, Chinese Academy of Sciences, 320 West Donggang Road, Lanzhou, 730000 China; 5 University of Chinese Academy Sciences, 19(A) Yuquan Road, Shijingshan District, Beijing, 100049 China

## Abstract

There are several publications related to the soil organic carbon (SOC) on the Qinghai-Tibetan Plateau (QTP). However, most of these reports were from different parts of the plateau with various sampling depth. Here, we present the results from a systematic sampling and analysis of 200 soil pits. Most of the pits were deeper than 2 m from an east-west transect across the plateau. The SOC and total nitrogen (TN) pools of the 148 × 10^4^ km^2^, the area of the permafrost zone, for the upper 2 m soils calculated from the vegetation map were estimated to be 17.07 Pg (interquartile range: 11.34–25.33 Pg) and 1.72 Pg (interquartile range: 1.08–2.06 Pg), respectively. We also predicted the distribution of land cover types in 2050 and 2070 using decision tree rules and climate scenarios, and then predicted SOC and TN pools of this region. The results suggested that the SOC and TN pools will decrease in the future. The results not only contribute to the carbon and nitrogen storage and stocks in the permafrost regions as a whole but most importantly, to our knowledge of the possible changes of C and N storage on the QTP in the future.

## Introduction

Soil carbon and nitrogen cycles are associated with terrestrial ecosystem succession and climate change. Small changes in the SOC pool could have dramatic impacts on the concentration of CO_2_ in the atmosphere^1^. Soil nitrogen is a key element controlling terrestrial ecosystems, and the decomposition of nitrogen may also produce an important greenhouse gas^2^. Spatial data for SOC and TN are extremely important because they are basic input parameters for many models, including land surface, vegetation, and greenhouse gas models^3–5^.Therefore, the size and dynamics of carbon and nitrogen pools are of great importance for recognizing the relationships among global change, terrestrial ecosystems, and the carbon cycles.

About half of the global SOC is stored in the permafrost regions^6^. Several studies on the SOC and TN in circum-Arctic permafrost regions^7–9^ indicated that large amount of greenhouse gases may be emitted from the decomposition of soil organic matter (SOM) in these regions due to permafrost degradation^10,11^. The largest high altitudinal permafrost on earth is distributed in the Qinghai-Tibetan Plateau (QTP), which is called ‘the Roof of the World’. Continuous permafrost underlies 1.06 × 10^6^ km^2^ of the permafrost area, and the total permafrost-affected zone^12^, including the discontinuous and island permafrost zone, is about 1.48 × 10^6^ km^2 ^^13^. Due to the low latitude, degradation of the permafrost in this area has received more attention^14^ because the degradation processes are more pronounced than in high latitude regions^15^.

The cold and arid grassland is the main ecosystem in permafrost zone on the QTP, and much more carbon is stored in soils than that in vegetation^16^. These soils contain a high proportion of labile SOM, which is mainly mineralizable^17,18^. A modelling study using the CENTURY (version 4.5) suggested that the SOC pools on the QTP, especially for the high temperature permafrost zones, are sensitive to environmental changes^19,20^. Under global warming scenarios, permafrost degradation might lead to the land degradation and a rapid loss of labile SOM, and further affect the permafrost ecosystems^21^ as well as carbon cycle^22,23^.

Previous publications on SOC pools on the QTP were estimated mostly based on the data from either global soil database or China’s national soil survey, combined with sparse field samples^24,25^. Total SOC for the top 0.7 m was estimated as approximately 30–40 Pg in the grassland of the plateau with the area of 1.67** × **10^6^ km^2^, which is much larger than the extent of permafrost zone^24^. The SOC pool in the permafrost regions was calculated as 17.3 ± 5.3 Pg for the 0–1 m depth and 10.6 ± 2.7 Pg for the 1–2 m depth based on similar database^26^. However, the filed data used in these studies were mainly collected from the upper 1 m depth or even less than 1 m depth, and at limited numbers of sites in the permafrost regions. The most recent result showed that the SOC pool in the upper 3 m soils was 15.31 Pg (with interquartile range of 13.03–17.77 Pg) based on a dataset from 342 soil cores^27^. The median and mean elevation of these cores was 4292 and 4124 m asl, while the average value of the lower limit of permafrost is higher than 4400 m asl^14^. In addition, these estimations were based on the 1:1,000,000 vegetation map of China^28^, which defined the land cover types as alpine steppe, meadow, and desert, without the consideration of wet meadow or the barren land. In fact, wet meadow and barren land constitute 3.4% and 19% of the total area, respectively^29^, and the soils under these two land cover types have very different SOC and TN stocks compared to other land cover types^30,31^.

Increasing temperature will accelerate decomposition of SOM. On the other hand, it can enhance primary production as if the soil moisture met the requirement of plant growth^32^. Since permafrost degradation alters the soil temperature and moisture conditions^33^, even forms thermokarst terrains, such as thaw slump and thermokarst lake^34–36^, it would significantly affect the SOC pools^37–40^. Despite the uncertainties in the changes of SOM along with the climate, the close relationship between land cover and SOC and TN stocks has been widely recognized^6,33,41,42^. We hypothesized that such relationship could be used to estimate the changes of SOC and TN storage along with the changes of land cover types.

During 2009–2013, we performed a large-scale field-sampling programme covering representative permafrost zones from the bioclimatic gradient of the QTP, including a large unpopulated area with harsh natural conditions. A total of 200 soil pits (pedons) were excavated; most of these pits were deeper than 2 m. The median and mean elevation of these sampling sites were 4467 and 4521 m asl (supplementary materials dataset 1). Based on this dataset, we calculated the SOC and TN stocks at specific soil layers (0–30 cm, 30–50 cm, 50–100 cm, and 100–200 cm). Then, we used a recently updated vegetation map (including the wet meadow and barren land in permafrost zone on the QTP) to assess the SOC and TN pools^29^. Finally, we projected the possible changes in SOC and TN pools according to changes of land cover using decision trees and the bioclimatic data for 2050 and 2070 provided by IPCC5. The aim of this study was to provide information about SOC and TN pools in this permafrost region based on our original data and to show their possible changes in the future.

## Results

### SOC and TN pools

**Figure 1:**
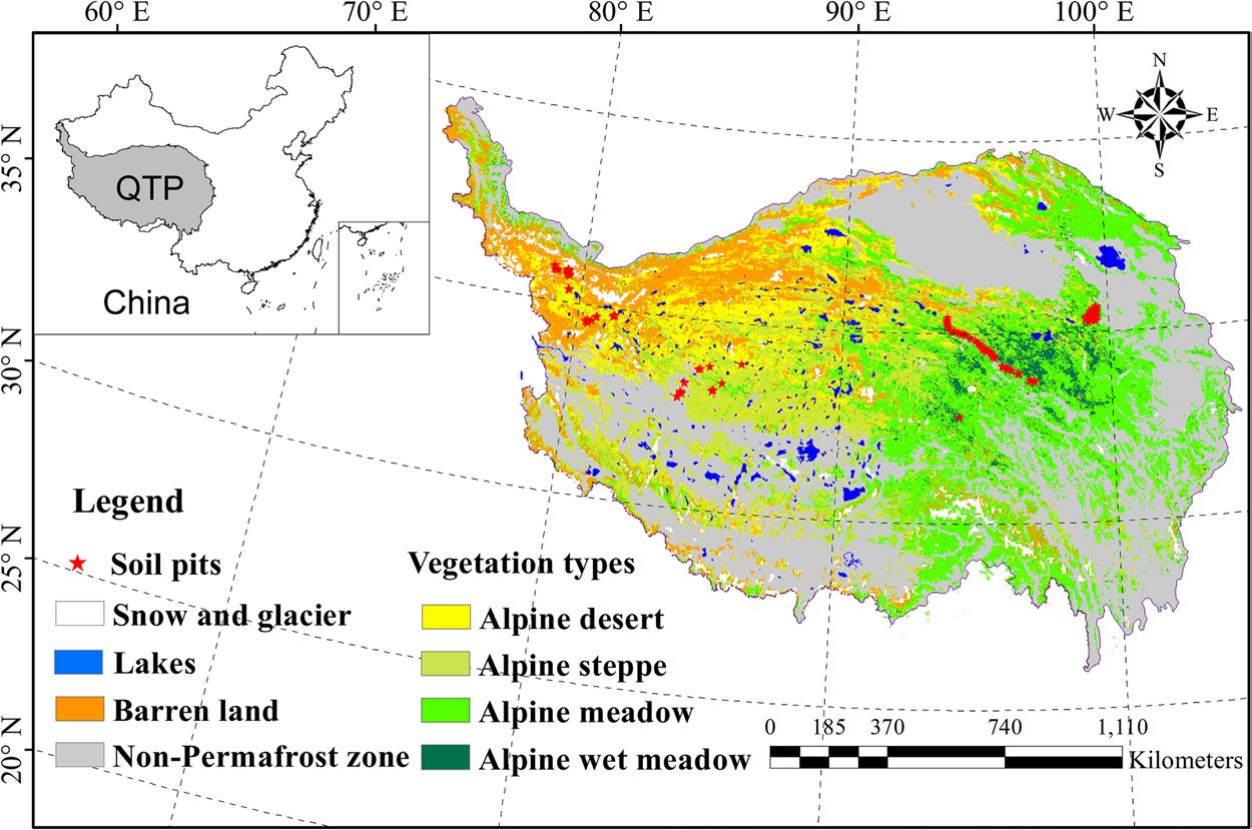
Distribution of soil pits and land cover types in the permafrost zone of Qinghai-Tibetan Plateau by using ArcGIS 9.3. The dataset of land cover types in permafrost regions of Qinghai-Tibetan Plateau was cited from the literature of Wang *et al*.^29^. The grey area is non-permafrost zone and was not studied.

**Figure 2:**
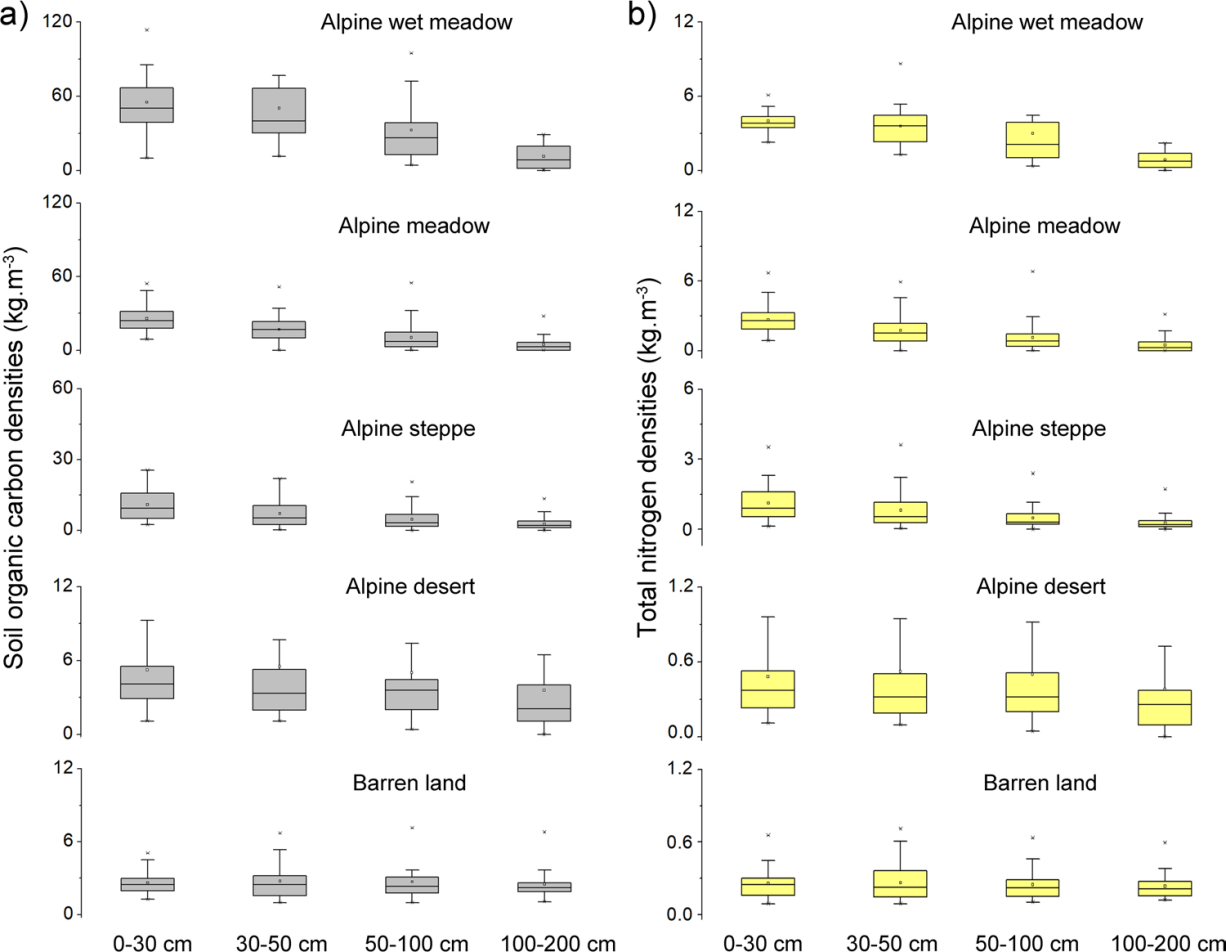
Soil organic carbon (**a**) and total nitrogen densities (**b**) for different layers in the permafrost regions on the Qinghai-Tibetan Plateau. The boxes are shown as 25th, 75th percentiles, and the lines within the boxes show the median values. The small boxes are the mean values. The whiskers indicate the 90th and 10th percentile, and the asterisks indicate the outliers.

According to the SOC and TN stocks at different depths and sites (Supplementary Table 1), we calculated the SOC and TN densities for the different layers. The alpine wet meadow (AWM) and alpine meadow (AM) were mainly distributed in the eastern part, whereas the alpine desert (AD) and barren land (BL) were distributed in the western part of the plateau (Fig. 1). The highest SOC densities occurred in the soils of AWM at the 0–30 cm layer (51.57 kg m^−3^), followed by the AWM at 30–50 cm (40.19 kg m^−3^). In the soils under alpine steppe (AS), the SOC densities at different layers were about half of those of meadow. For the soils of BL, the SOC densities were the lowest. SOC densities largely decreased with depth, except for the soils of BL, which showed similar values at different layers. The TN densities showed similar patterns to those of SOC, i.e., the highest TN densities were recorded in the soils under AWM, and the lowest values under BL (Fig. 2).

The vegetation map of the QTP permafrost zone covered an area of 148 × 10^4^ km^2^. The AWM, AM, and AS accounted for 3.4%, 39.3%, and 22.4%, respectively. The AD and BL covered 15.8% and 19.0% of the total area, respectively. Using these areas and the SOC and TN stocks at different depths, the SOC and TN storages could be calculated according to land cover types (Table 1). For the different layers, both the SOC and TN showed similar spatial patterns (Supplementary materials dataset 1). The uncertainty ranges for SOC and TN stocks increased markedly along with the depth, indicating that the SOC and TN stocks varied greatly. Overall, the SOC and TN storages for the upper 200 cm soils in the permafrost regions on the QTP were 17.07 Pg (interquartile: 11.34–25.33 Pg) and 1.72 Pg (interquartile: 1.08–2.06 Pg), respectively. The median values of C/N ratios ranged from 8.13 to 15.00 (with an average value of 12 for all the median values) in the upper 200 cm soils under all land cover types. The upper soil layers (0–50 cm) showed higher values than lower soil layers.

**Table 1:** SOC and TN stocks and storages (median values with interquartile range) in permafrost zones of the QTP under different land cover types.

Depths	Vegetation	SOC (kg/m^2^)	TN (kg/m^2^)	C/N ratios	SOC storage (Pg)	TN storage (Pg)	Total SOC (Pg)	Total TN (Pg)
0–30 (cm)	AWM	15.48 (14.15–17.09)	1.17 (1.11–1.21)	15.00 (14.62–15.61)	0.78 (0.73–0.86)	0.06 (0.05–0.06)	6.05 (5.74–6.39)	0.61 (0.58–0.65)
	AM	7.25 (6.84–7.43)	0.77 (0.71–0.81)	10.39 (10.23–10.68)	3.81(3.60–3.99)	0.41(0.39–0.43)		
	AS	2.85 (2.77–2.91)	0.27 (0.25–0.34)	9.97 (9.83–10.09)	0.95 (0.92–0.97)	0.09 (0.09–0.11)		
	AD	1.27 (1.13–1.45)	0.12 (0.10–0.13)	10.71 (10.33–11.02)	0.30 (0.28–0.35)	0.03 (0.03–0.03)		
	BL	0.74 (0.73–0.76)	0.07 (0.06–0.08)	10.42 (10.11–11.25)	0.21 (0.21–0.22)	0.02 (0.02–0.02)		
30–50 (cm)	AWM	8.04 (6.21–13.12)	0.73 (0.46–0.88)	14.94 (12.99–15.70)	0.41 (0.31–0.66)	0.04 (0.02–0.04)	2.81 (2.14–3.73)	0.26 (0.20–0.32)
	AM	3.33 (2.98–3.58)	0.30 (0.28–0.33)	10.05 (9.66–10.27)	1.74 (1.47–1.92)	0.15 (0.14–0.16)		
	AS	1.07 (0.51–2.08)	0.16 (0.06–0.23)	9.34 (9.17–9.67)	0.36 (0.17–0.69)	0.04 (0.02–0.08)		
	AD	0.70 (0.40–1.17)	0.06 (0.04–0.11)	10.20 (10.05–10.95)	0.16 (0.10–0.28)	0.02 (0.01–0.02)		
	BL	0.50 (0.31–0.64)	0.04 (0.03–0.07)	10.36 (9.44–12.53)	0.14 (0.09–0.18)	0.01 (0.01–0.02)		
50–100 (cm)	AWM	14.82 (7.03–19.01)	1.12 (0.53–1.90)	12.76 (10.64–14.96)	0.745 (0.36–0.96)	0.06 (0.03–0.10)	3.86 (1.84–7.10)	0.39 (0.19–0.71)
	AM	3.49 (1.33–7.35)	0.41 (0.19–0.71)	8.99 (7.22–11.75)	1.80 (0.70–4.04)	0.21 (0.08–0.40)		
	AS	1.60 (0.82–3.36)	0.16 (0.10–0.33)	10.26 (7.60–11.47)	0.53 (0.28–1.12)	0.05 (0.03–0.11)		
	AD	1.90 (1.05–2.30)	0.17 (0.12–0.27)	10.10 (8.59–11.75)	0.45 (0.25–0.54)	0.04 (0.03–0.06)		
	BL	1.16 (0.89–1.54)	0.11 (0.07–0.14)	11.00 (9.79–12.16)	0.33 (0.25–0.44)	0.03 (0.02–0.04)		
100–200 (cm)	AWM	9.04 (2.57–19.16)	0.82 (0.25–1.34)	12.97 (9.84–16.02)	0.46 (0.13–0.97)	0.04 (0.01–0.07)	3.57 (1.30–6.66)	0.35 (0.12–0.71)
	AM	2.59 (0.05–6.19)	0.26 (0.01–0.73)	8.13 (6.56–11.76)	1.23 (0.02–2.71)	0.13 (0–0.34)		
	AS	1.99 (1.07–3.82)	0.19 (0.10–0.36)	9.25 (7.60–13.79)	0.66 (0.36–1.27)	0.06 (0.03–0.12)		
	AD	2.56 (1.11–4.15)	0.26 (0.18–0.42)	8.59 (7.86–10.95)	0.60 (0.26–0.97)	0.06 (0.04–0.10)		
	BL	2.21 (1.89–2.63)	0.21 (0.15–0.27)	11.29 (9.65–12.50)	0.62 (0.53–0.74)	0.06 (0.04–0.08)		
0–200 (cm)	AWM	53.03 (26.27–66.12)	3.83 (2.19–5.53)	13.39 (11.45–15.15)	2.67 (1.32–3.32)	0.20 (0.11–0.28)	17.07 (11.34–25.33)	1.72 (1.08–2.06)
	AM	17.49 (11.37–25.23)	1.83 (1.21–2.78)	9.90 (7.96–11.50)	8.79 (6.38–14.14)	1.00 (0.60–1.49)		
	AS	7.66 (4.80–13.13)	0.76 (0.52–1.35)	9.73 (8.45–11.42)	2.55 (1.60–4.26)	0.25 (0.17–0.44)		
	AD	7.40 (4.08–8.94)	0.63 (0.45–1.01)	9.89 (8.62–11.54)	1.74 (0.96–2.10)	0.15 (0.11–0.24)		
	BL	4.65 (3.80–5.34)	0.41 (0.33–0.54)	10.67 (9.89–12.82)	1.32 (1.08–1.51)	0.12 (0.09–0.15)		

The detailed information of the SOC and TN stocks for each pit was shown in supplementary information (dataset S1). AWM: Alpine wet meadow (50260 km^2^); AM: Alpine meadow (583909 km^2^); AS: Alpine steppe (332754 km^2^); AD: Alpine desert (234828 km^2^); BL: Barren land (282657 km^2^). The land cover types were defined according to the field evidence including vegetation cover and the dominant species.

### Changes of SOC and TN pools in 2050 and 2070

Through analysis of the bioclimatic data using the decision tree rules, the land cover types showed different changes in areas (Supplementary material dataset 2); and the changes of SOC and TN for different climate scenarios derived from the different GCMs varied considerably at 2050 and 2070 (Supplementary material Figs S1–S4). Most of the results suggested that the SOC and TN pools would decrease under four representative concentration pathways (RCPs), although those from HadGEM2-ES and MRI-CGCM3 suggested increases of SOC and TN pools for 2050 and 2070. For 2050 and 2070, 72% and 72.5% of the GCMs showed decrease in the SOC pools, and 62.5% and 67.5% of the GCMs showed decrease in the TN pools.

**Figure 3:**
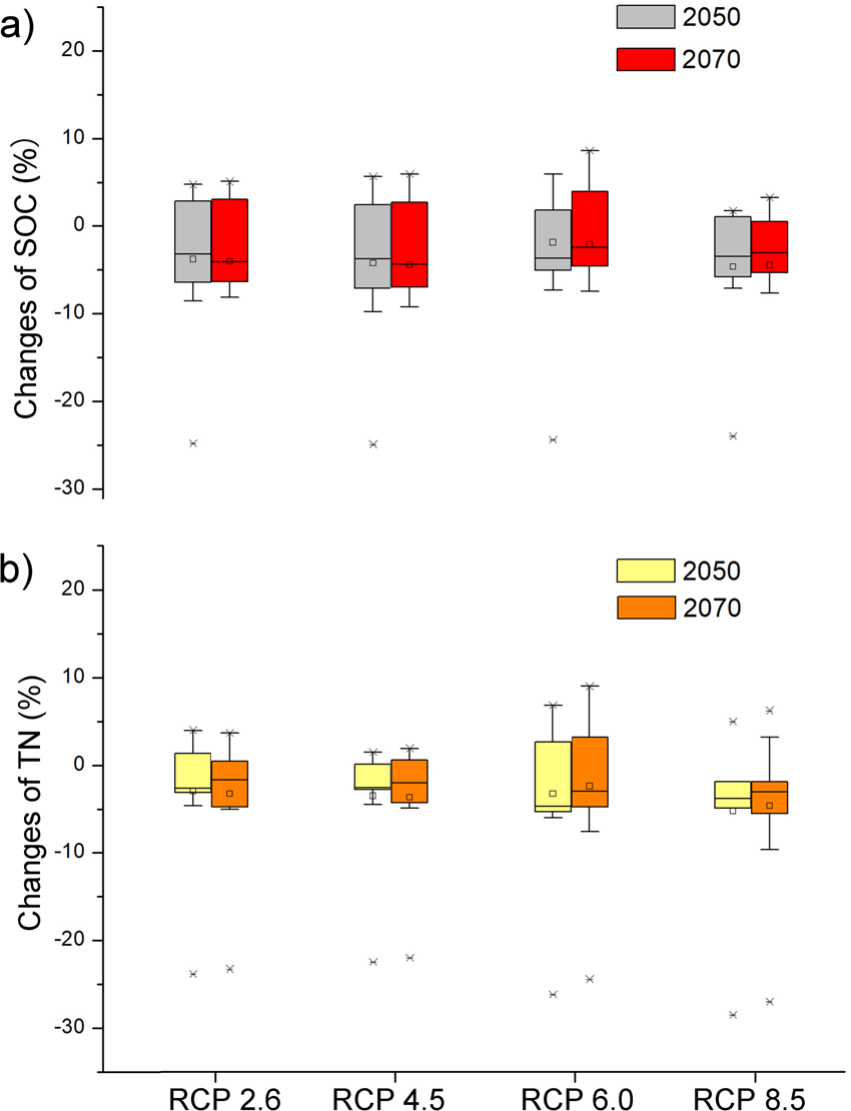
Changes of (**a**) soil organic carbon and (**b**) total nitrogen pools (%) according to land cover changes under different emission scenarios. The boxes are shown as 25th, 75th percentiles, and the lines within the boxes show the median values. The small boxes are the mean values. The whiskers indicate the 90th and 10th percentile, and the asterisks indicate the outliers.

The statistical results of the changes of SOC and TN storages from the GCMs are shown in Fig. 3. The SOC under the four RCPs decreased by approximately 3% for both 2050 and 2070. TN pools showed a decrease of approximately 2%.

## Discussion

Although altitude affects the distribution of SOC on the QTP^43^, the land cover types have the most important effects on SOC since the altitude has been included as an input parameter in the mapping of the land cover on the QTP^29^. The AWM and AM contained over 10 times the SOC and TN stocks of BL, and 4 to 5 times those of AD and AS. The SOC and TN stocks were significantly different among land cover types, which fit the pattern of land cover types affecting the SOM density^42,44^. The AWM, which is usually accompanied by high SOM due to high primary production and favourable conditions for perseverance of SOM such as high moisture content^45^, only covers small areas in the eastern permafrost region, while it contributes large amounts of SOC relative to its covering area. The SOC stocks for AM and AD were comparable to those in previous reports on the northern and eastern part of the permafrost regions in the QTP^33,46^.

The circum-Arctic regions largely have high SOC stocks (15–50 kg.m^−2^) for 0–1 m soils^47^. The lower SOC stocks on the QTP could be explained by the pedogenesis, vegetation cover, moisture, and permafrost conditions. The QTP is overall characterized by a low vegetation cover and weak soil development^48^, leading to much lower carbon accumulation rates than that in circum-Arctic regions. In the western QTP that is mainly covered by AS and BL, the climate is arid and cold^49^, which results in thin A horizons and without O horizons. The eastern part of QTP is characterized by a subhumid climate. Therefore, the O and A horizons are thicker than the western QTP^50^. In our study, several soil pits reached to the bedrock within 2 m, and the SOC and TN stocks were only calculated within the solum layers. This caused the greater uncertainties in deeper soils (Table 1), suggesting that it is extremely important to map the soil thickness on the QTP in the future. In addition to the weak soil development, the QTP is considered as “warm” permafrost and the active layers are usually deeper than 2 m except the AWM that has permafrost tables shallow than 2 m^51,52^. Thus, it would be reasonable to expect that the active layers are too deep to affect the SOM perseverance in the upper part of the soils^53^, which is a possible explanation for the lower SOC and TN stocks for the QTP. In many soils, the SOC and TN showed similar spatial distribution patterns under different land cover types^54^. In our study, the highest and lowest stocks of TN were observed in AWM and BL soils.

The permafrost carbon pools on the QTP for the upper 2 m soils were 2~3% of that in circum-Arctic regions, whereas the permafrost region in the QTP was approximately 8% of that in circum-Arctic regions^47^. Since the AM is mainly distributed in the eastern part of the QTP, the eastern QTP area stores approximately 80% of the SOC. The biomass and net primary production is high in eastern but much lower in the western part^55^. The main reason for the spatial distributions of SOC and land cover types is the annual precipitation. The higher precipitation favours to vegetation growth and development of permafrost, subsequently leading to high SOC and TN contents.

There are some reports regarding the SOC pools on the QTP and even the permafrost regions on the QTP^24,27,56^. However, it is difficult to cross-reference or compare the results and estimate SOC pools at specific depth intervals since previous studies were based on data from in which the SOC was measured with different methods (dichromate or loss on ignition) and different sampling depths. In the present study, sampling at all of the sites was conducted using the same procedure, and the SOC pools at different depths were provided at a resolution of 1 km × 1 km. We also provided the spatial distribution of permafrost N stocks and pool of QTP. This result forms a useful database for research such as land-surface processes modelling.

In the upper 1 m soils, the median values of C/N ratios showed decreasing trends with depth in different land cover types^57^. We found the C/N ratios decreasing from the upper to lower soil layers in many pits (Table 1 and supplementary materials dataset 1), which indicates an increased degree of decomposition^58,59^. For the different land cover types, the highest mean values appeared at different depths. This was caused by the high SOM content in some pits where cryoturbation existed as in most AWM and AM soils; these layers usually had high contents of SOC with low decomposition rates and large weights in the calculation of the mean C/N ratios. The C/N ratios for different vegetation types showed a similar pattern to that of SOM content, indicating that SOM stored in the AWM had the lowest decomposition rate, followed by that in the AM and AS. The BL showed higher C/N ratios than those of AS and AD, which could be explained by the fact that the BL was mainly distributed in higher altitude regions characterized by lower temperature and thus lower microbial decomposition rates. The BL areas were usually higher than 4900 m asl, whereas the AWM, AM, AS and AD were usually distributed in the areas between 4600–4800 m asl.

The predicted losses of SOC and TN in permafrost regions will be 3% and 2%, respectively, till 2070 in assumption of that the relationship between land cover type and SOM content is unchanged under global warming scenarios. In fact, global warming significantly affects the SOM contents via multiple processes. The rising temperature can increase the decomposition of SOM. Meanwhile, climate warming can enhance plant growth and increase primary production, and thus provide more input of carbon to soils^5^. In permafrost regions, this process is more complicated because there is the interaction between SOM and permafrost: permafrost can prevent the decomposition of SOM and translocate the surface SOM to deeper soil by cryoturbation^60,61^. On the QTP, deepening of active layer can decrease soil water content in upper soils and further decrease vegetation biomass^62^. Therefore, permafrost degradation affects the litter input to soils, which is a main pathway for the SOM formation. In this study, we primarily showed the possible changes of SOC and TN according to the land cover. Due to the great differences in bioclimatic data produced by different GCMs, the predicted land cover types varied greatly (supplementary material dataset S2), but the predicted SOC and TN storage showed overall decreasing trends under global warming scenarios. This result was in agreement with the findings from experimental warming in permafrost ecosystems^63–65^.

In conclusion, the present study estimates the SOC and TN stocks and storages in permafrost regions of the QTP using data from soil pits. For all land cover types, the SOC and TN pools for the upper 2 m were 17.07 Pg (interquartile range: 11.34–25.33 Pg) and 1.72 Pg (interquartile range: 1.08–2.0 6 Pg), respectively. There are wide uncertainties in the SOC and TN storages in the permafrost zone on the QTP because the soil thicknesses varied greatly under different topographic conditions. The SOC and TN were mainly distributed in the eastern part of the plateau. Approximate 51.5% of SOC and 58.1% of TN were stored in the soils under the meadow. Based on bioclimatic data provided by the IPCC5, the total SOC and TN pools would decrease under global warming scenarios. These results will update the global carbon and nitrogen database and provide a basis for further research on global warming and greenhouse emission, as well as ecology succession studies.

## Methods

### Sampling and Analysis

The field work was performed between 2009 and 2013. A total of 200 soil pits were excavated. Seventy-two soil pits were manually dug in 2009, and 128 soil pits were excavated using a hydraulic excavator (Doosan 331) in 2010 and 2011. In our study, the active layer thickness is deeper than 2 m at most of sampling sites, but there were still some pits encountered the permafrost within 2 m. For these sites, we used a diesel rig to drill boreholes and collected the permafrost samples. Most of the pits were deeper than 2 m, unless rock layers were detected. As shown in Fig. 1, the sampling sites were distributed both in eastern and western parts of the plateau. It has been shown that SOC stocks in permafrost areas varied greatly with depths and soil samples should be collected according to soil horizons^66^. On the QTP, the soils have weak pedogenesis and the organic layers were only found in the AWM areas^30,45^. In the eastern part, where the soil horizons were thinner than the depth increments, we collected soil samples according to the soil horizons and then calculated the SOC and TN stocks for different depths. For the soil pits which had simple horizons, we collected soil samples at depth intervals as 0–10 cm, 10–20 cm, 20–30 cm, 30–50 cm, 50–100 cm, and 100–200 cm. The entire profiles were collected using a spade. At each depth, at least 1 kg soils (dried weight) were collected from the bottom layers to upper layers. The SOC and TN pools according to depth interval were reported in this study. In addition, since many permafrost areas on the QTP have active layers deeper than 2 m, we did not separate the SOC and TN pools in the active layers and permafrost layers. The land cover types were defined according to the field evidence including vegetation cover and the dominant species. These study areas covered the typical land cover types and permafrost conditions (different active layer thickness, different ground temperature) on the QTP. The vast area of the plateau could be divided into eastern and western parts, according to the land cover types. The eastern part was predominantly alpine meadow and wet meadow, whereas the western was alpine steppe and desert steppe. According to precipitation data from the China Meteorological Administration (http://data.cma.cn/data/cdcdetail/dataCode/SURF_CLI_CHN_MUL_DAY_V3.0.html), the eastern parts were characterized by a sub-humid climate and the western part was composed of arid and semi-arid regions. Thus, our sampling sites could be representative of the eastern and western parts of the plateau. In fact, our dataset probably reached the maximum representation possibility in this area under present conditions because most parts of the permafrost regions are considered “no man’s land,” with harsh natural conditions and limited road access.

For most soils, the field bulk density was determined using metal-ring method. For the permafrost samples, which were collected from the drilling method, the bulk density was measured using core sampler method^67^. The rock fragment (mineral particles > 2 mm) content was calculated from the oven-dried samples in a field laboratory using a sieving method. The SOC and TN were measured with K_2_Cr_2_O_7_—H_2_SO_4_ and the micro-Kjeldahl method, respectively^31^.

### Vegetation map

The areas which were underlain by permafrost are smaller than the areas of permafrost zone since permafrost free areas are common in the discontinuous and sporadic zones (https://ipa.arcticportal.org/publications/occasional-publications/what-is-permafrost). In our study, we used the areas of permafrost zone on the QTP since the permafrost carbon in the circum-arctic regions refers to the areas of the permafrost zone^47^. The most updated vegetation map for the permafrost regions on the QTP^29^ was applied in the present study. In short, a decision tree classification algorithm was constructed using the field monitoring data to classify alpine grassland types with the statistical software See 5.0 (http://www.rulequest.com). The results from analysis of the EVI (Enhanced Vegetation Index, MOD13A2, 2009–2013), LST (Land Surface Temperatures, MOD11A2, 2009–2013), and DEM (Digital Elevation Model) datasets with 1 km × 1 km resolution, which were obtained from NASA (National Aeronautics and Space Administration) (https://ladsweb.modaps.eosdis.nasa.gov/ or ftp://ladsweb.nascom.nasa.gov/) and the EESDC (Environmental and Ecological Science Data Centre for West China) (http://westdc.westgis.ac.cn/), were used to build the vegetation map. The new vegetation map contained more detailed information about the vegetation distribution than the 1:1,000,000 vegetation map of China^28^ and also included the wet meadow and barren land types^29^. Based on the rules of this decision tree, the vegetation distributions in 2050 and 2070 were mapped using the bioclimatic data (BIO1-BIO19) provided by IPCC 5 (http://www.worldclim.org/cmip5_30s). Bioclimatic data from GCMs, including BCC-CSM1-1,CCSM4, GISS-E2-R, HadGEM2-AO, HadGEM2-ES, IPSL-CM5A-LR, MIROC-ESM, MIROC5, MRI-CGCM3, and NorESM1-M, were selected to predict the changes in land cover areas and to calculate the SOC and TN pools. Note that the areas of the land cover types from the current bioclimatic data have some differences with that we used to report the SOC and TN storages. We reported the SOC and TN storages based on the land cover types from Wang *et al*. (2016) because this map is more accurate by the validation^29^. We used the land cover areas from the current bioclimatic data to predict the future changes of SOC and TN storages since the data were produced by same procedure and thus it is possible to predict the changes in the areas of different land cover types. The land cover areas used in the GCMs were shown in supplementary materials dataset 2.

### SOC and TN calculation

All of the analyses were carried out in triplicate using subsamples. Total SOC stocks per square metre were calculated as:

1 $$P=\sum {r}_{i}{H}_{i}{b}_{i}(1-{g}_{i})\times {10}^{-3}$$

where *P* (kg m^−2^) was the SOC stock at the given soil layer, *r* was the soil bulk density (kg m^−3^), *H* was the mean soil layer thickness (m) and *b* was the mean organic carbon content (g kg^−1^), g was the rock fragment content (%), and *i* was the depth interval being analysed. The stock of TN was determined using the same calculation but by substituting TN in place of SOC content. The SOC and TN stocks were calculated for following intervals: 0–30 cm, 30–50 cm, 50–100 cm, and 100–200 cm.

The data are presented as median values. The uncertainty analysis was conducted using the Percentile Method, and we used a quartile confidence interval to find the range of SOC and TN stocks and storages for different soil layers. All the data analysis was performed using R statistical software (version 3.3.3, www.r-project.org).

## Electronic supplementary material

Supplementary information

Dataset 1

Dataset 2
