# Supplementary material for: Soil organic carbon and total nitrogen pools in permafrost zones of the Qinghai-Tibetan Plateau
Source: Sci Rep. 2018 Feb 26;8:3656. doi: 10.1038/s41598-018-22024-2 (PMC5827726; doi:10.1038/s41598-018-22024-2)

**Soil organic carbon and total nitrogen pools in permafrost zones of the Qinghai-Tibetan Plateau**

**Author names:** Lin Zhao^1^, Xiaodong Wu^1^, Zhiwei Wang^1,2^, Yu Sheng^1^, Hongbing Fang^1^, Yonghua Zhao^1^, Guojie Hu^1^, Wangping Li^3^, Qiangqiang Pang^1^, Jianzong Shi^1^, Bentian Mo^2^, Qian Wang^2^, Xirui Ruan^2^, Xiaodong Li^2^, Yongjian Ding^1^

**Affiliation**: ^1^ Cryosphere Research Station on the Qinghai-Tibetan Plateau, State Key Laboratory of Cryospheric Science, Northwest Institute of Eco-Environment and Resources, CAS, Lanzhou, 730000, China

^2^ Guizhou Institute of Prataculture, Guizhou Academy of Agricultural Sciences, Guiyang, 550006, China

^3^ School of civil engineering, Lanzhou University of Technology, Lanzhou, 730050, China

**Corresponding authors:** LinZhao, Xiaodong Wu

**Corresponding address:** Cryosphere Research Station on the Qinghai-Tibetan Plateau, State Key Laboratory of Cryospheric Science, Northwest Institute of Eco-Environment and Resources, CAS, West Road of Donggang, Lanzhou, 730000, China

Tel: +869314967717 (L.Z.) or +8618909408575 (X.W.), E-mail: [linzhao@lzb.ac.cn](mailto:linzhao@lzb.ac.cn) (L. Z.) or [wxd565@163.com](mailto:wxd565@163.com) (X.W.)

Data set 1 Environmental factors and soil variables at different depths at different soil sites. The geographical coordinate, elevation, aspect, gradient, vegetation cover, land cover type, stock, content, density, storage of SOC and TN, gravel content, and bulk density were shown in the dataset. The land cover types were defined according to the field evidence including vegetation cover and the dominant species.

Data set 2 Vegetation cover (%) under different models for current, 2050 and 2070 using bioclimate data.

Figure S1: Changes of SOC (%) for different GCMs in four RCPs in 2050.


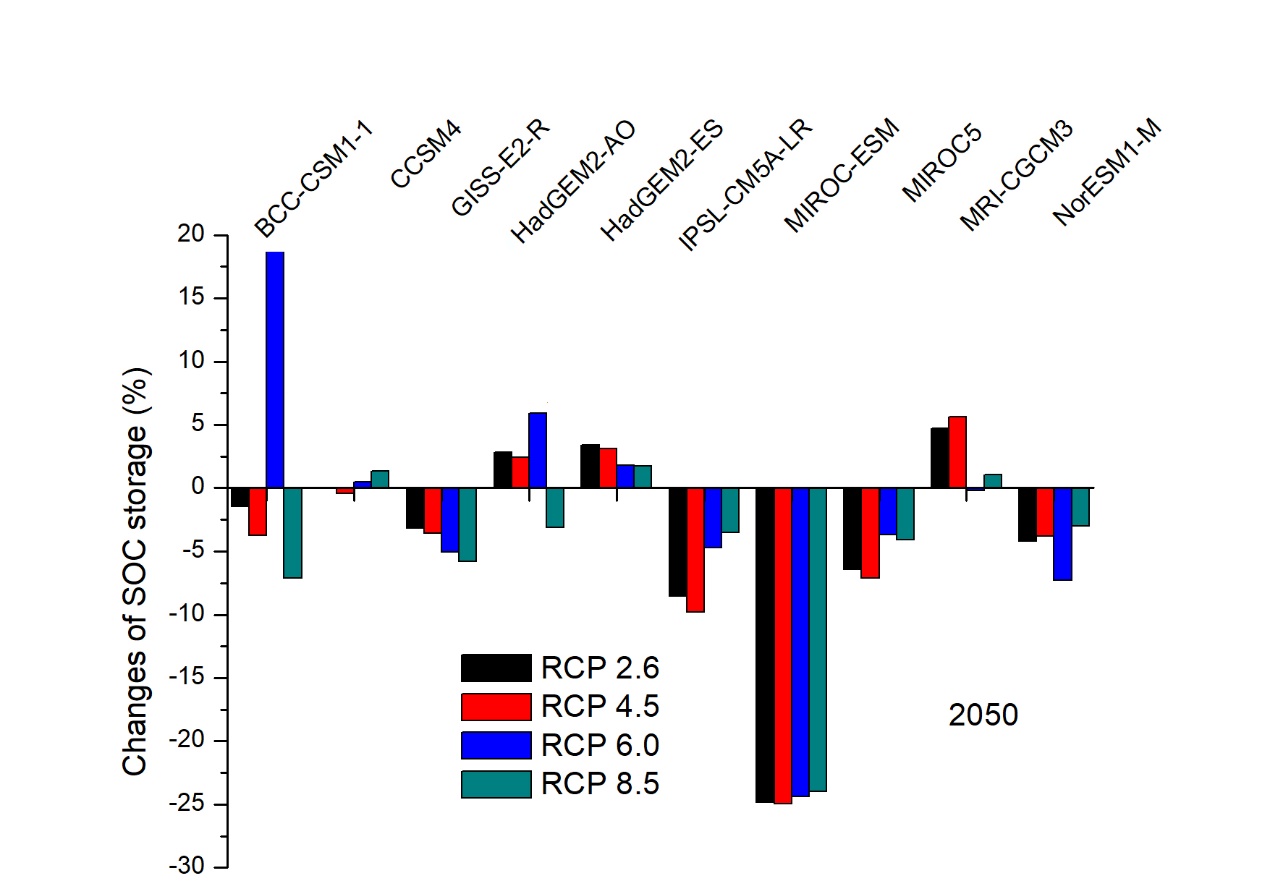


Figure S2: Changes of SOC (%) for different GCMs in four RCPs in 2070.


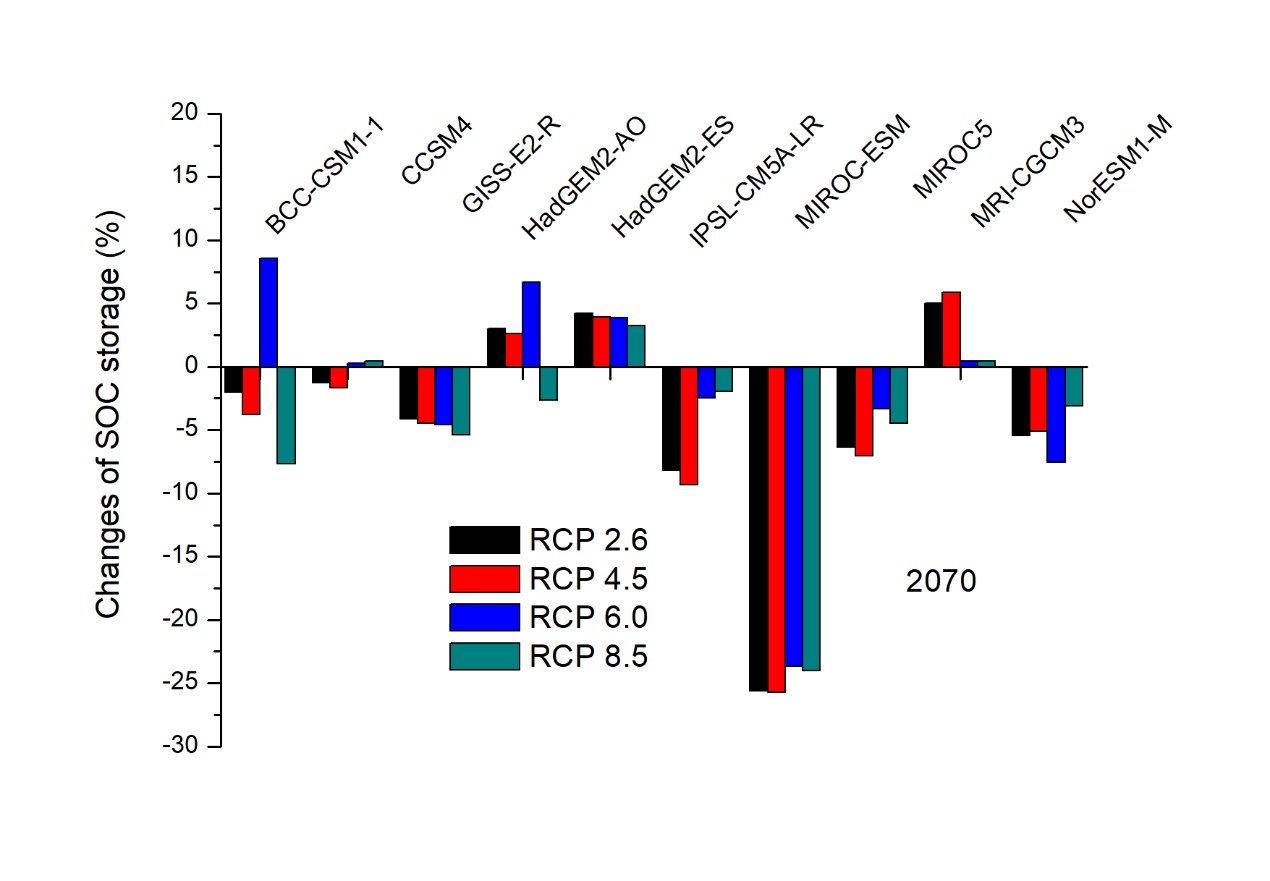


Figure S3: Changes of TN (%) for different GCMs in four RCPs in 2050.


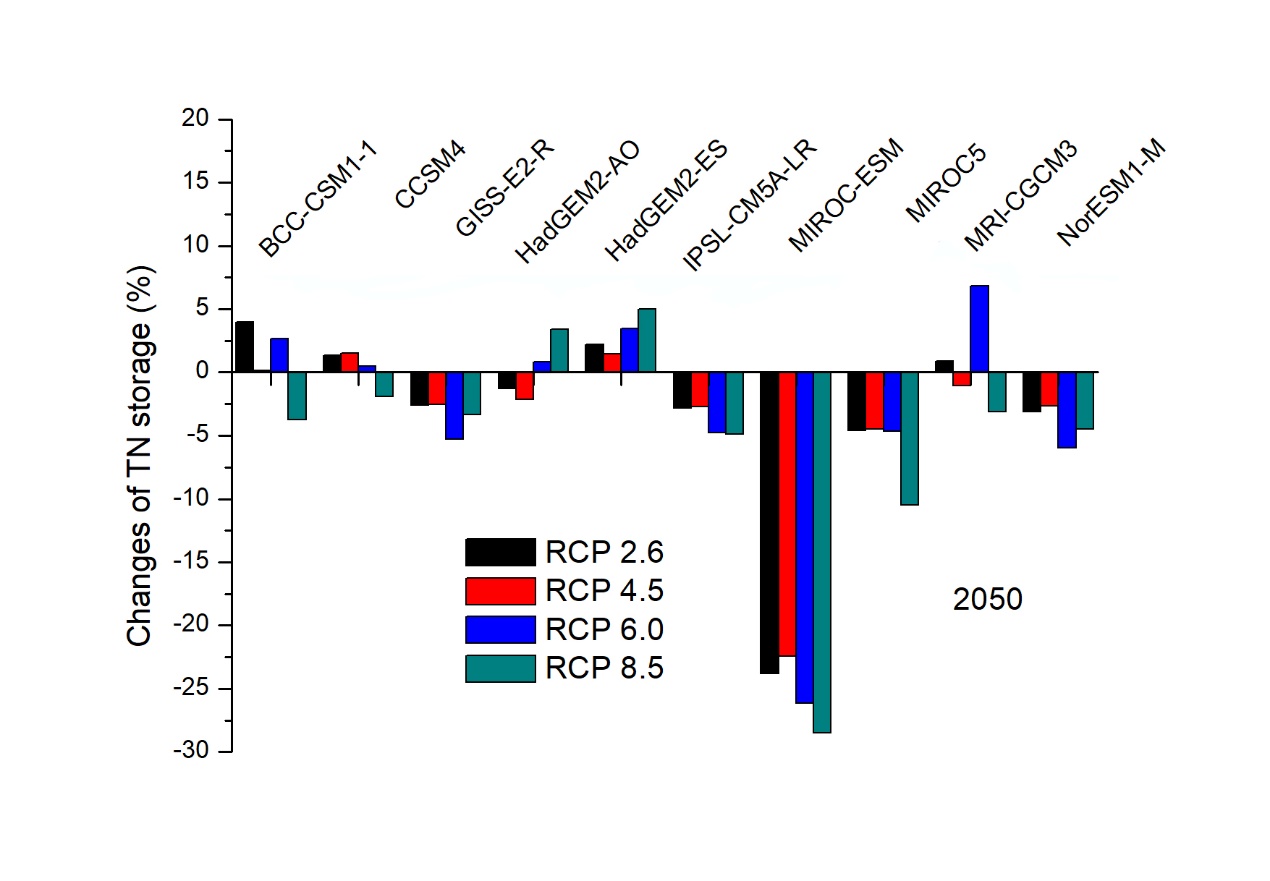


Figure S4: Changes of TN (%) for different GCMs in four RCPs in 2070.


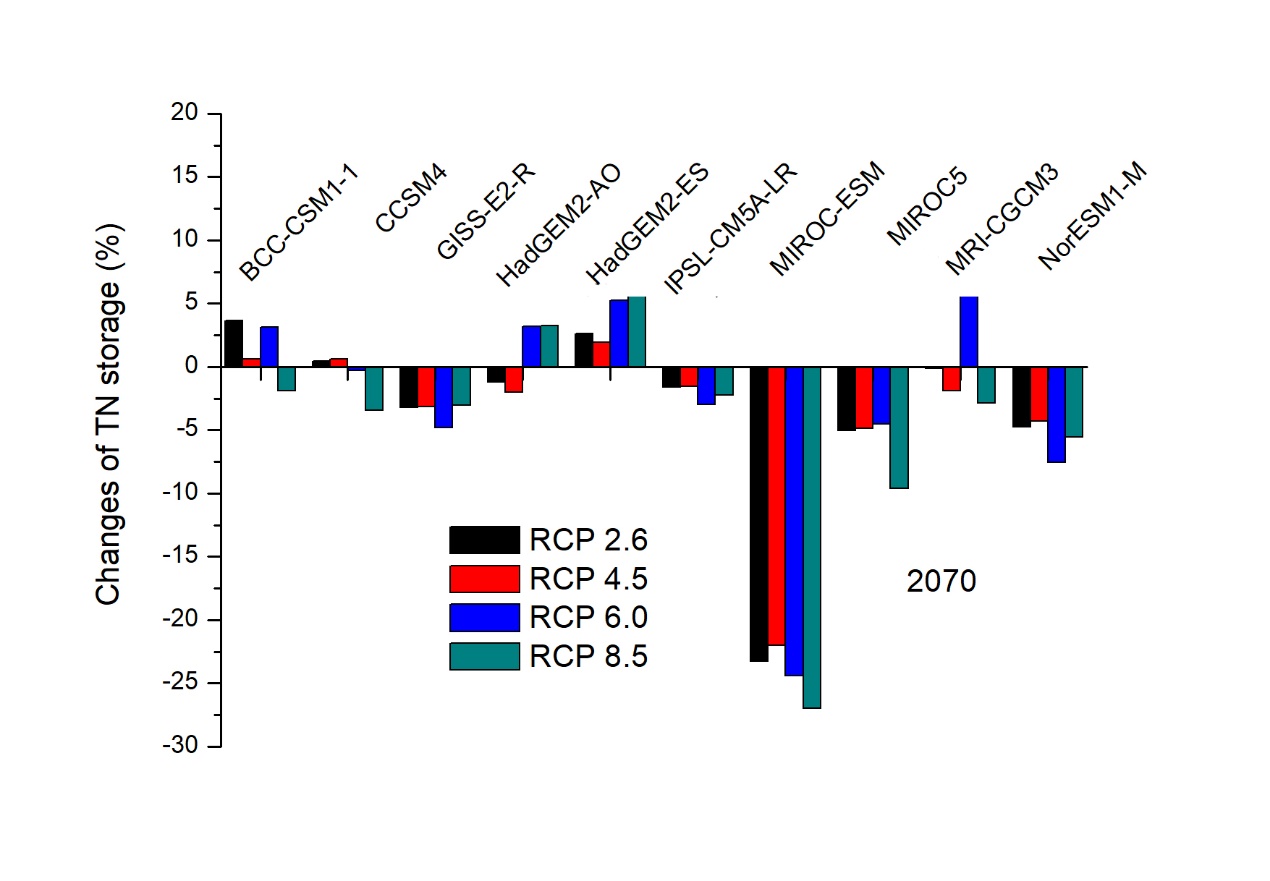

Supplement: Supplementary file 1 — Supplementary information [file 41598_2018_22024_MOESM1_ESM.doc]
